# Supplementary material for: Dye Plants Derived Carbon Dots for Flexible Secure Printing
Source: Nanomaterials (Basel). 2022 Sep 13;12(18):3168. doi: 10.3390/nano12183168 (PMC9506027; doi:10.3390/nano12183168)
Supplement: Supplementary file 1 [file nanomaterials-12-03168-s001.zip › nanomaterials-1868632-supplementary.pdf]

nanomaterials

Supplementary Materials

Dye Plants Derived Carbon Dots for Flexible Secure Printing

Linlin Li [1], Yuanyuan Han [1], Lihua Wang [2], Wei Jiang [1,*] and Haiguang Zhao [2,*]

[1] College of Textiles & Clothing, Qingdao University, No. 308 Ningxia Road, Qingdao 266071, China
[2] State Key Laboratory of Bio-Fibers and Eco-Textiles, College of Physics, Qingdao University, No. 308 Ningxia Road, Qingdao 266071, China
* Correspondence: weijiangqd@qdu.edu.cn (W.J.); hgzhao@qdu.edu.cn (H.Z.)

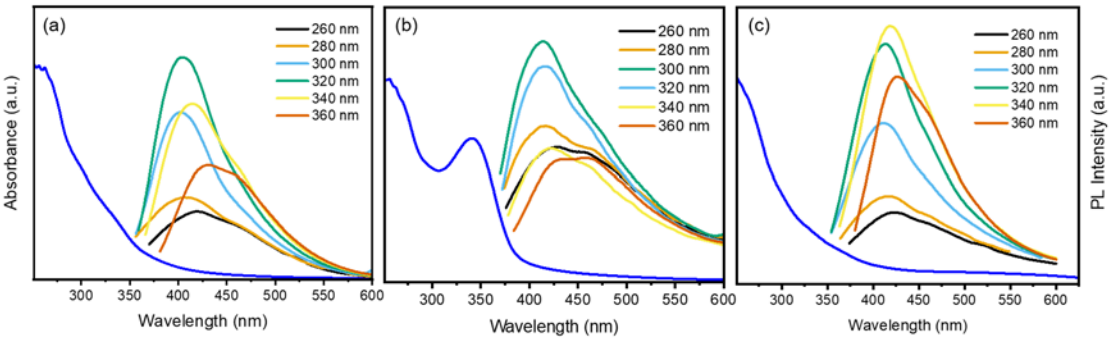

Figure S1. Absorption and PL spectra of the C-dots prepared using water as solvent (**a**): Indigo C-dots; (**b**): *Carcuma longa* C-dots; (**c**): *Sophora japonica* L. C-dots.

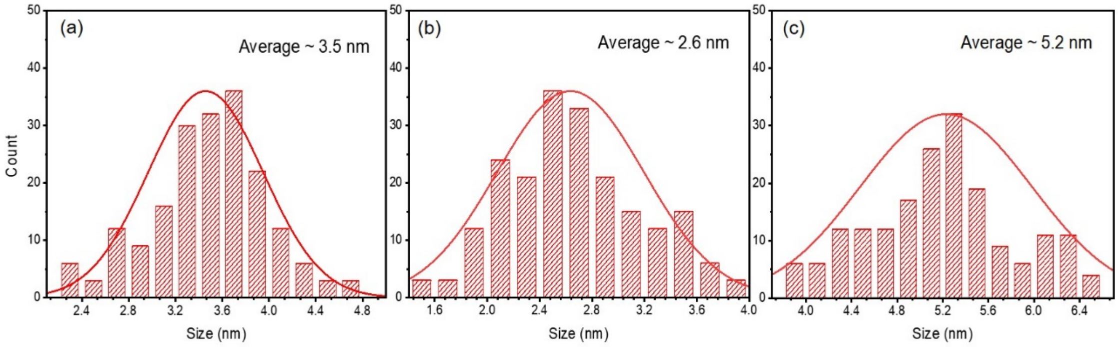

Figure S2. Diameter distribution of C-dots. (**a**): Indigo C-dots; (**b**): *Carcuma longa* C-dots; (**c**): *Sophora japonica* L. C-dots.

Table S1. Quantitative analysis results of XPS data for C-dots derived from different natural dyes.

Sample	C (%)	O (%)	N (%)	Cl (%)	Si (%)	S (%)	Se (%)
Indigo C-dots	71.98	17.24	3.89	3.32	2.95	0.62	0
Carcuma longa C-dots	78.54	17.21	1.68	0.46	1.61	0.49	0
Sophora japonica L. C-dots	64.47	26.56	5.92	2.89	0	0	0.17

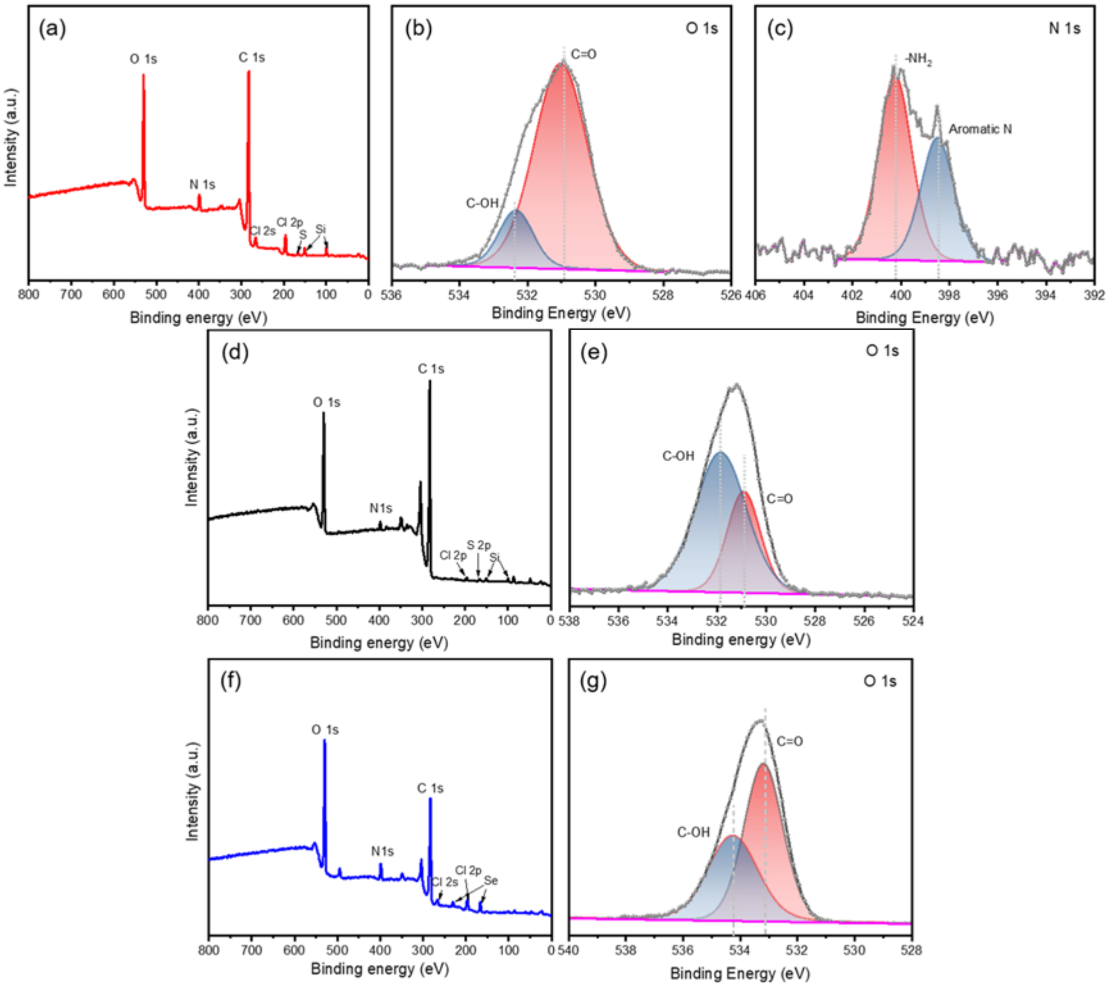

Figure S3. XPS full survey and high resolution XPS spectra. The C-dots were prepared using (**a**,**b**,**c**) Indigo, (**d**,**e**) *Carcuma longa*, and (**f**,**g**) *Sophora japonica* L.

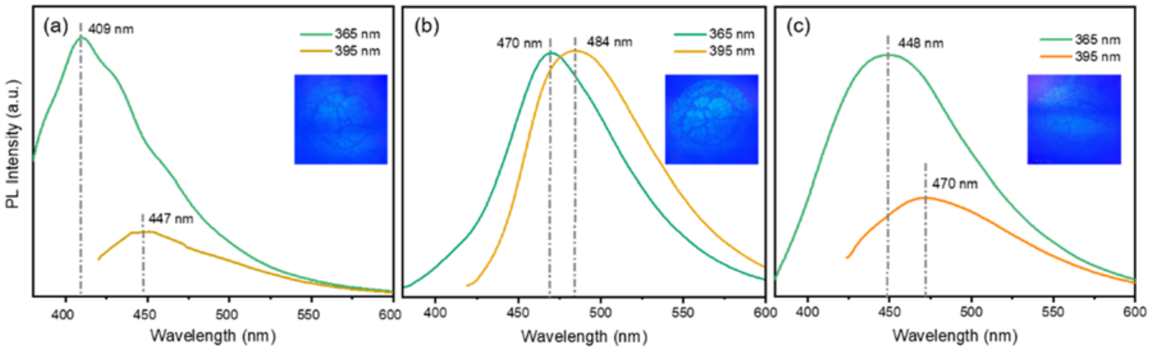

Figure S4. PL spectra of C-dots inks and printed patterns on folded flexible fabrics. The C-dots were derived from (**a**) Indigo; (**b**) *Carcuma longa* and (**c**) *Sophora japonica* L. Insets: the folded pattern on flexible cotton fabric under 395 nm illumination.

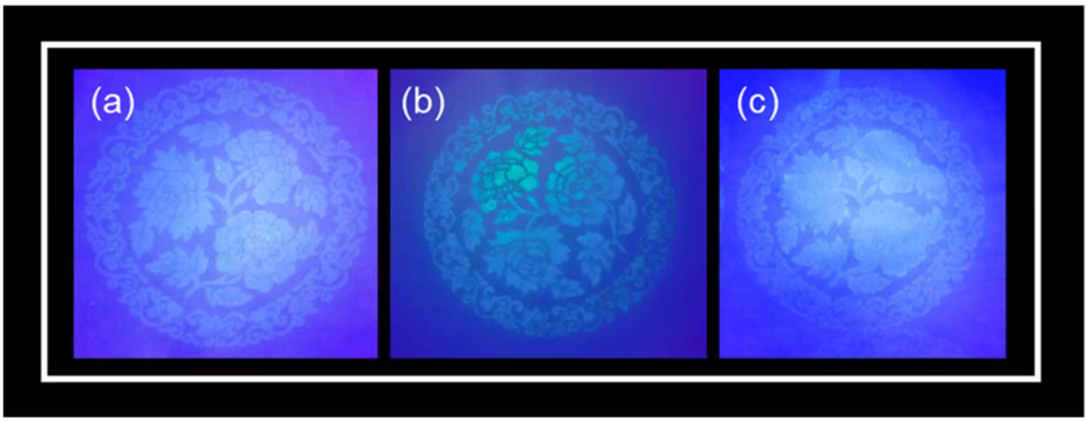

Figure S5. The printed anti-counterfeit patterns on cotton fabric after 3 months of storage upon 395 nm illumination. The C-dots were prepared using (**a**) Indigo, (**b**) *Carcuma longa*, and (**c**) *Sophora japonica* L.

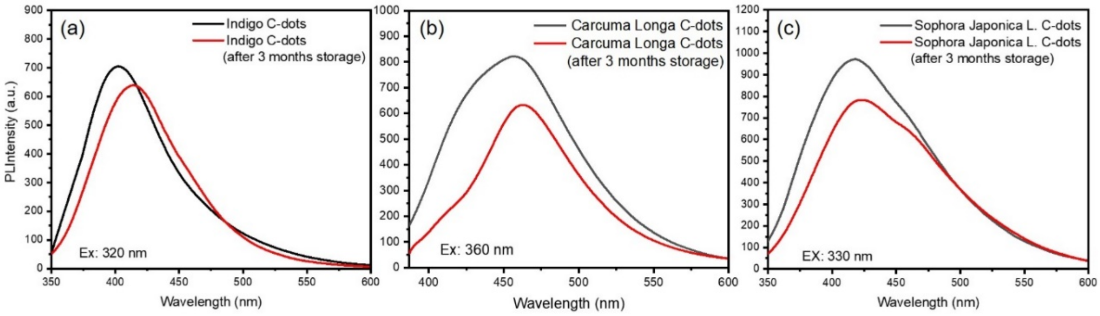

Figure S6. PL spectra of C-dots solution after 3 months of storage. The C-dots were prepared using (**a**) Indigo, (**b**) *Carcuma longa*, and (**c**) *Sophora japonica* L.

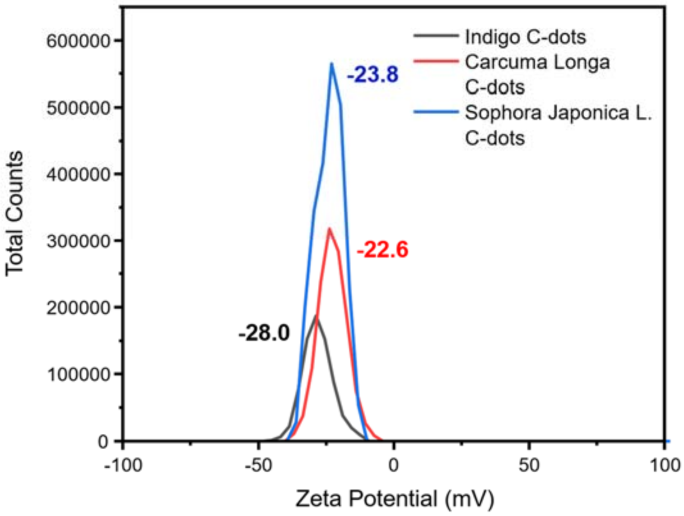

Figure S7. Zeta potential of C-dots derived from indigo, *Carcuma longa* and *Sophora japonica* L.
